# Choosing a treatment method for post-catheterization pseudoaneurysms guided by the late to early velocity index

**DOI:** 10.1038/s41598-021-96062-8

**Published:** 2021-08-17

**Authors:** Jacek Kurzawski, Lukasz Zandecki, Agnieszka Janion-Sadowska, Lukasz Piatek, Anna Jaroszynska, Szymon Domagala, Marcin Sadowski, Edyta Baranska

**Affiliations:** 1 Swietokrzyskie Cardiology Center, ul. Grunwaldzka 45, 25-736 Kielce, Poland; 2 Jan Kochanowski University, Collegium Medicum, al. IX Wiekow Kielc 19A, 25-317 Kielce, Poland

**Keywords:** Interventional cardiology, Cardiology, Medical research

## Abstract

Ultrasound-guided thrombin injection (UGTI) is often the first-line treatment for iatrogenic post-catheterization pseudoaneurysms (psA). There are also first reports of the use of biologically derived tissue glues (TG) instead of sole thrombin especially when UGTI was unsuccessful or in case of psA recurrence. Previously, we have established that a late to early velocity index (LEVI) < 0.2 could be a predictor of an increased risk of psA recurrence after standard UGTI. In this paper, we report our first experiences when the choice of the first-line treatment method was based on LEVI assessment. From May 2017 till January 2020 we included 36 patients with psA. Of them, 10 had LEVI < 0.2 and they underwent ultrasound-guided tissue glue injection (UGTGI) with biological TG and 26 had LEVI > 0.2 and they underwent UGTI. The injection set containing human thrombin and fibrinogen was used for UGTGI. Bovine thrombin was used for UGTI. The success rate was 100% and no psA recurrence was detected during a 2-week follow-up. It was significantly better when compared to the expected recurrence rates based on our previous 14 years of experience (0% vs. 13%, *p* = 0.01). All complications (10% in the UGTGI group and 15% in the UGTI group) were mild and transient and included clinical symptoms of paresthesia, numbness, tingling, or pain. Their rates were comparable to the rates we previously reported. No significant differences in other characteristics were observed. The approach to choose the first-line treatment method for iatrogenic psA based on LEVI is encouraging. It may increase the success rate and avoid unnecessary repetition of the procedure, without increasing complication rate while keeping costs of the procedure reasonable.

## Introduction

Iatrogenic pseudoaneurysms (psA) complicate about 1.5% of the vessel cannulation procedures^1^. Ultrasound-guided thrombin injection (UGTI) is a commonly performed procedure for the treatment of psA^2^. The reported overall success rate of UGTI has been over 97%^1–4^. However, the procedure needs to be repeated at least once in up to around 13% of cases due to psA recurrence^2,4^.

Tissue glues (TG) are currently divided into two groups: synthetic and biologically derived. The use of N-butyl-cyanoacrylate for psA embolization has been reported^5,6^. However, degradation products of synthetic TG may have a toxic effect on living cells^7–9^ which may raise a concern about the safety of injecting it directly into psA which communicates with patient’s circulation. The group of biologically derived TG is represented by fibrin glues that consist of a fibrinogen solution only or in combination with thrombin. They act immediately upon application by augmenting local hemostasis and they are widely used in surgery as a topical tissue sealant. We reported the effectiveness of biologically derived TG instead of sole thrombin for psA embolization in selected patients after unsuccessful UGTI^10^. Furthermore, we named this technique ultrasound-guided tissue glue injection (UGTGI). UGTGI is a similar technique to UGTI. The main advantage of UGTGI is the simultaneous application of both thrombin and fibrinogen, which rapidly interact and create a stiff thrombus. Theoretically, the use of TG may increase the procedure success rate at the cost of a potentially higher risk of complications in the case of too much the substance escape into the patient’s circulation during the procedure. TG is also more expensive than thrombin, so it is typically reserved for the use in specific conditions when UGTI is unsuccessful or in case of psA recurrence.

It may not be necessary to use UGTGI in all patients as a first-line treatment method but there is no validated parameter to distinguish between patients who are likely to benefit from the stronger procoagulant properties of TG and those who can be effectively treated with standard UGTI. We have previously proposed late to early velocity index (LEVI) as a novel parameter to indicate patients with psA that may be prone to recurrence^3^. LEVI is a simple parameter derived from the analysis of the spectral Doppler waveform across the neck of the psA during the outflow phase. Following precise analysis of the waveform, we hypothesize that early and late velocities are not arranged randomly. We observed that LEVI depended on the psA size or the stage of clot formation and retraction. Patients with low LEVI values were those whose psA had a higher flow and lower resistance flow across the neck and they were more prone to psA recurrence. A LEVI value of less than 0.2 has been identified a significant predictor of a psA recurrence^3^.

The study was aimed at evaluating if the choice of the first-line treatment method of psA (UGTGI or UGTI) based on LEVI assessment can provide satisfactory outcomes in terms of the procedure success rate andpsA recurrence rate.

## Methods

This is a prospective non-randomized historically controlled cohort study. From May 2017 till January 2020 we included 36 consecutive patients with psA. There were no specific exclusion criteria. The patients were hospitalized in a single Cardiology Centre in Poland. They underwent transcatheter cardiac procedures. For femoral procedures arterial sheaths removal was followed by manual compression for 15–20 min with subsequent mechanical compression with a pressure dressing for six hours. For radial procedures dedicated hemostasis bands were used. Once a patient was diagnosed with psA and qualified for transcutaneous psA treatment, the method was chosen based on LEVI. If LEVI was < 0.2 the patient underwent UGTGI. Otherwise, the patient was treated with standard UGTI. The analyzed outcomes included the success of the procedure, recurrence within 2 weeks and any reported complications. The patients and psA characteristics as well as the outcomes were compared between both UGTGI and UGTI groups. The recurrence and complications rates were also compared with the historical cohort of 508 patients with psA treated with UGTI in our center between 2004 and 2017 (in this cohort the observed recurrence rate was 15% and the observed complication rate was 14%)^3^.

**Figure 1:**
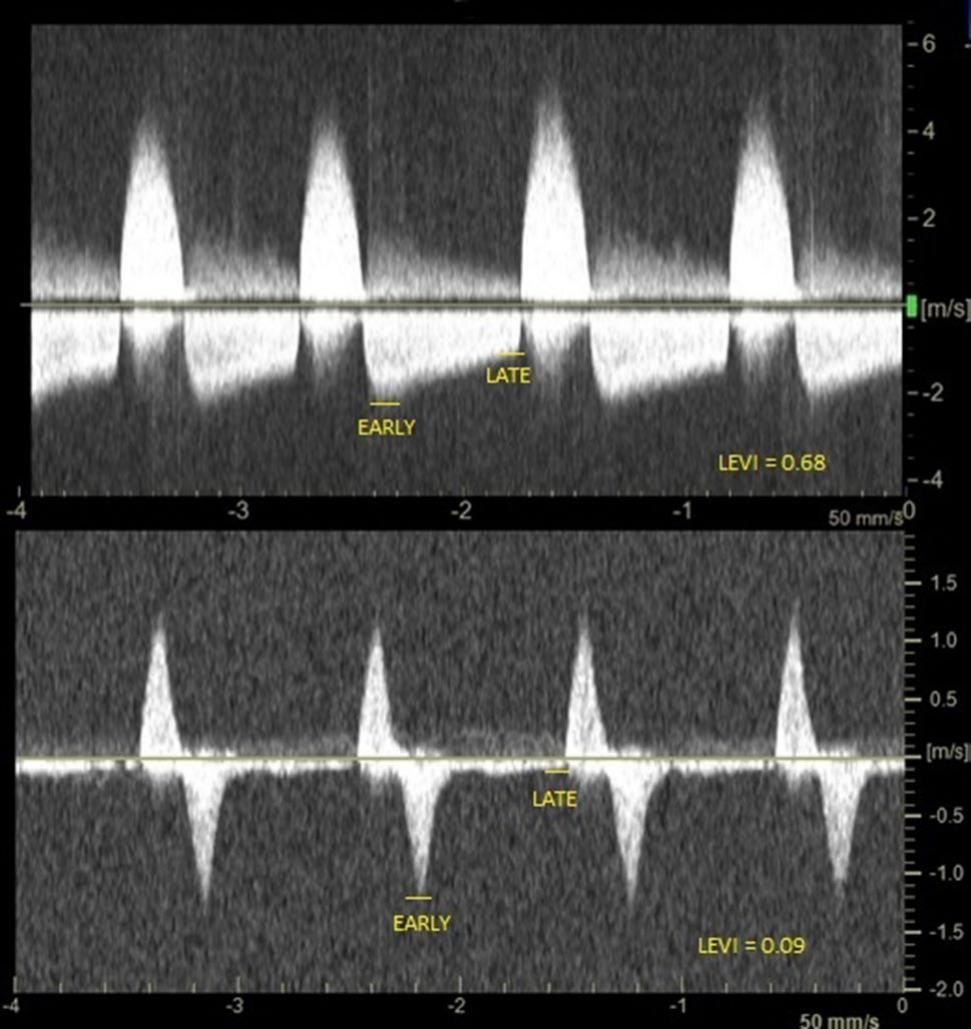
The examples of late to early velocity index (LEVI) calculations.

PsA were visualized using General Electric Vivid E9 and E95 machines with linear 7–10 MHz and sector 2.5–3.5 MHz ultrasound probes. Data were acquired using two-dimensional imaging and color Doppler mapping, blood flow across the neck of psA was assessed using a pulsed wave or continuous wave spectral Doppler. Analysis of the psA structure included the presence and length of a communicating channel (neck), approximate psA volume calculated in milliliters using an ellipsoid formula 4/3 π (a × b × c), where a, b, and c represented radii along each axis^3,4^. LEVI was analyzed in the outflow Doppler spectrum during diastole, between two consecutive systolic inflow phases^3^. The LEVI was calculated by dividing the late outflow velocity (just before the beginning of the inflow phase) by the early outflow velocity (just after the beginning of the outflow phase). The examples of LEVI calculations are presented in Fig. 1.

**Figure 2:**
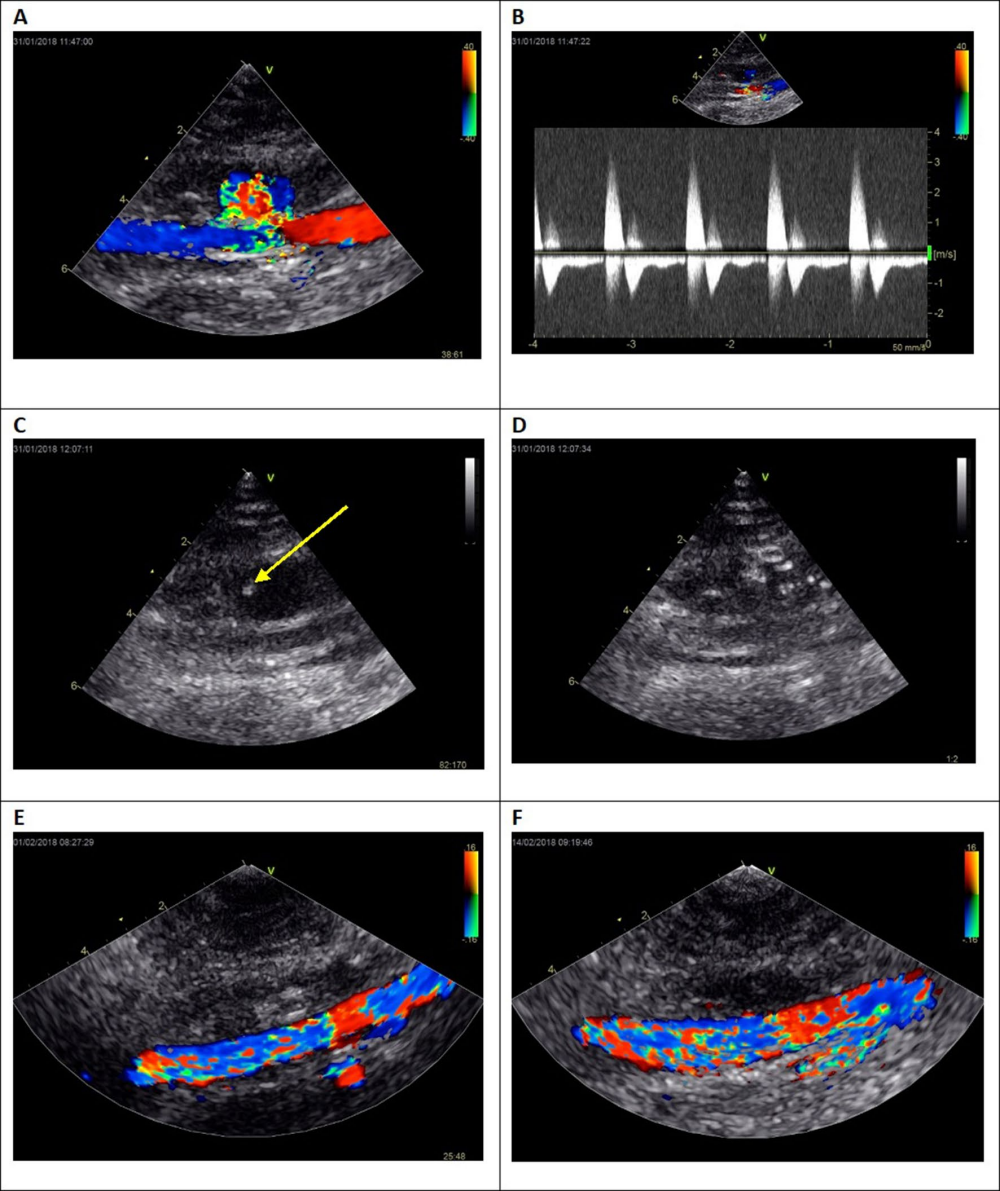
The example of the UGTGI procedure. (**A**) Ultrasound two-dimensional longitudinal image with color Doppler before psA embolization. (**B**) Pulse-wave Doppler image of inflow and outflow from the sac of psA, LEVI = 0.18. (**C**) Inserting a needle into the psA sac. The arrow indicates the tip of the needle. (**D**) Injection of TG into the psA sac. (**E**) Ultrasound image after the embolization of psA. (**F**) Follow-up ultrasound examination after 2 weeks. psA—pseudoaneurysm, UGTGI—ultrasound-guided tissue glue injection, LEVI—late to early velocity index, TG—tissue glue.

Both UGTI and UGTGI techniques were previously described in detail^4,10^. In brief, the injection set (Tisseel Lyo, Baxter AG, Vienna, Austria), containing human thrombin and fibrinogen, was used for UGTGI. After preparation according to manufacturer instructions, two solutions were drawn into 2 ml syringes which were then placed in a special delivery device enabling simultaneous injection from the two syringes. Bovine thrombin (“Thrombin 400” and “BioThrombin 400,” Biomed Wytwornia Surowic i Szczepionek Spolka z o.o., Lublin, Poland) was used for UGTI. The needle was advanced under ultrasound guidance, and positioned in the patent psA sac, as far from the psA inflow orifice as possible. During and after each application of the drug, psA thrombosis and blood flow were observed using USG imaging and Doppler. In case of unsuccessful complete psA thrombosis or residual blood flow in the psA, another fraction of substance was injected during the same procedure. All procedures were performed by one experienced operator. Follow-up ultrasound was performed 24 h later, and then at 1 and 2 weeks after the procedure. Figure 2 illustrates the example of the UGTGI procedure. The UGTI procedures were performed in the same way except for the substance used for embolization.

All patients involved were informed about the procedure and written consent was acquired before the treatment. All procedures were performed in accordance with the ethical standards and with the 1964 Helsinki declaration and its later amendments. The use of TG for the treatment of psA has been approved by the Bioethics Committee of the Swietokrzyska Chamber of Physicians in Kielce.

Categorical data were presented as counts and percentages. Pearson’s chi-square test was used to compare categorical data with Yates correction applied if any expected frequency was < 5. Quantitative data were presented as mean and standard deviation or median and interquartile range depending on data distribution. Mann–Whitney U test was used to compare quantitative data. A *p*-value < 0.05 was considered statistically significant. Statistical analyses were performed using Statistica (data analysis software system), version 13. TIBCO Software Inc. (2017).

## Results and discussion

Of 36 patients included in the study, 10 had LEVI < 0.2 and they underwent UGTGI and 26 had LEVI > 0.2 and they underwent UGTI. The comparison of UGTGI and UGTI groups is presented in Table 1.

**Table 1:** Comparison of UGTGI and UGTI groups.

	All (n = 36)	UGTGI (n = 10)	UGTI (n = 26)	*p*-value
Late to early velocity index—LEVI	0.42 ± 0.27	0.15 ± 0.04	0.52 ± 0.24	< 0.001
Age (years)	72 ± 9	72 ± 7	71 ± 10	0.48
Female sex	26 (72%)	7 (70%)	19 (73%)	1
Time from cannulation (h)	59 (24–119)	89 (24–146)	52 (24–115)	0.54
Femoral artery	28 (78%)	9 (90%)	19 (73%)	0.4
Pseudoaneurysm volume (ml)	2.8 ± 3.1	1.9 ± 1.2	3.1 ± 3.6	0.78
Canal length (mm)	5.3 ± 6.4	5.5 ± 6.3	5.2 ± 6.6	0.97
Antiplatelets	28 (78%)	6 (60%)	22 (85%)	0.18
Antithrombotics	11 (31%)	3 (30%)	8 (31%)	1
Tissue glue dose (ml)	–	1.7 ± 1.4	–	–
Fibrinogen dose (mg)	–	78.3 ± 61.1	–	–
Thrombin dose (IU)^a^	110 (40–310)	450 (100–500)	60 (40–160)	< 0.001
Complications	5 (14%)	1 (10%)	4 (15%)	1

^a^ For UGTGI thrombin dose was calculated from the tissue glue amount used and the thrombin concentration in the tissue glue. UGTGI—Ultrasound-guided tissue glue injection, UGTI—Ultrasound-guided thrombin injection.

Most cases were femoral psA (superficial femoral artery—17 patients, common femoral artery—10 patients, deep femoral artery—1 patient) and the rest were psA of radial (7 patients including 1 patient in the UGTGI group) or brachial (1 patient) arteries. No significant differences in patients or psA characteristics were observed between the groups. The success rate was 100% and no psA recurrence was detected during a 2-week follow-up. It was significantly better when compared to the expected recurrence rates based on our previous 14 years of experience (0% vs. 15%, *p* = 0.01)^3^.

The median amount of thrombin used for UGTGI (which was calculated from the TG amount used and the thrombin concentration in the TG) was significantly higher than the median amount of thrombin used for UGTI. We followed the already tested protocol for UGTGI and the application technique was not guided by the thrombin amount^10^. We believe that it may not be an appropriate outcome measure to directly compare the amount of active substances in two different drugs used for the procedures because they have different pharmacodynamic properties and dosage protocols are not specifically based on the amount of thrombin in each drug.

### Complications

During the procedure 5 patients experienced mild and transient clinical symptoms of paresthesia/numbness/tingling (5 patients—14%) or pain (3 patients—8%). All those complications were minor conforming to the A category of the New Adverse Event Classification by the Society of Interventional Radiology^11^. Their rates were comparable to the rates we previously reported (14% vs. 14%, *p* = 0.96)^3^. No major complications were noted within 14-days of follow-up.

### Cost-effectiveness

During the study period, the local market price of one vial with 400 IU of bovine thrombin was approximately 10 Euro while 2 ml Tisseel Lyo set was around 10 times more expensive. In countries where only human thrombin is available the savings may be limited as the price difference is much lower. However, one important aspect needs to be considered when analyzing cost-effectiveness of the proposed treatment strategy—psA recurrence typically leading to prolonged hospitalization which generates further costs.

### The choice of treatment method for psA

Minimally invasive UGTI is commonly used as a first line treatment methods for iatrogenic psA. The role of surgery has diminished since the development of less-invasive methods but occasionally surgical repair is necessary^12,13^. In the past, techniques of thrombin embolization included transarterial injection or the additional use of balloon protection^12^ but nowadays it seems unnecessary as ultrasound-guided transcutaneous injection is safe and its success rates are high. It seems that to further improve already high success rates the choice of substances used for embolization is crucial. We have been using bovine thrombin and biological tissue glues, but the use of synthetic TG for psA embolization has also been reported^5,6^. Biodegradable adhesive bovine collagen was proposed as a substance effective for psA embolization and the experiences with its use were published by a single center in the Netherlands^14^. There are also institutions where ultrasound guided compression or even manual compression repair without continuous ultrasound control are preferred treatment methods^12,15^. However, these methods have lower success rates especially in anticoagulated patients and are limited by patient discomfort and long procedural times^12^. Other very rarely used methods include implantation of stent grafts, coil embolization or vascular plugs^4,12^.

### Limitations

There are no data on reproducibility of LEVI calculations across institutions. This is a relatively novel parameter but when compared with other CW Doppler-derived parameters we believe that reproducibility of LEVI should be high. Despite study duration of 33 months we could only enroll 36 patients in this study and this is explained by the relatively low incidence of iatrogenic psAs. Larger, multi-center studies are needed to provide even higher quality evidence to confirm our findings. The additional comparison with the historical cohort has limitations typical to such an approach—although the general basics of UGTI technique did not change, the equipment quality improved and operator’s experience increased.

## Conclusion

The strategy based on LEVI seems to be a promising approach to select the patients who are most likely to benefit from the use of TG. This can be achieved without generating unnecessary costs and potentially putting patients at a higher risk of complications in whom standard UGTI will most likely be sufficient treatment.
